# Impact of social integration on metabolic functions: evidence from a nationally representative longitudinal study of US older adults

**DOI:** 10.1186/1471-2458-13-1210

**Published:** 2013-12-20

**Authors:** Yang Claire Yang, Ting Li, Yinchun Ji

**Affiliations:** 1 Department of Sociology, Lineberger Comprehensive Cancer Center, Chapel Hill, NC, 27517, USA; 2 Center for Population and Development Studies, Renmin University of China, Beijing, 100872, China; 3 Carolina Population Center, University of North Carolina at Chapel Hill, 123 W. Franklin St., Chapel Hill CB#8120 27516, NC, USA

**Keywords:** Social integration, Social network size, Metabolic functions, Total cholesterol, High-density lipoprotein cholesterol, Glycosylated hemoglobin, Waist circumference, Blood pressure, Older adults

## Abstract

**Background:**

Metabolic functions may operate as important biophysiological mechanisms through which social relationships affect health. It is unclear how social embeddedness or the lack thereof is related to risk of metabolic dysregulation. To fill this gap we tested the effects of social integration on metabolic functions over time in a nationally representative sample of older adults in the United States and examined population heterogeneity in the effects.

**Methods:**

Using longitudinal data from 4,323 adults aged over 50 years in the Health and Retirement Study and latent growth curve models, we estimated the trajectories of social integration spanning five waves, 1998–2006, in relation to biomarkers of energy metabolism in 2006. We assessed social integration using a summary index of the number of social ties across five domains. We examined six biomarkers, including total cholesterol, high-density lipoprotein cholesterol, glycosylated hemoglobin, waist circumference, and systolic and diastolic blood pressure, and the summary index of the overall burden of metabolic dysregulation.

**Results:**

High social integration predicted significantly lower risks of both individual and overall metabolic dysregulation. Specifically, adjusting for age, sex, race, and body mass index, having four to five social ties reduced the risks of abdominal obesity by 61% (odds ratio [OR] [95% confidence interval {CI}] = 0.39 [0.23, 0.67], p = .007), hypertension by 41% (OR [95% CI] = 0.59 [0.42, 0.84], p = .021), and the overall metabolic dysregulation by 46% (OR [95% CI] = 0.54 [0.40, 0.72], p < .001). The OR for the overall burden remained significant when adjusting for social, behavioral, and illness factors. In addition, stably high social integration had more potent metabolic impacts over time than changes therein. Such effects were consistent across subpopulations and more salient for the younger old (those under age 65), males, whites, and the socioeconomically disadvantaged.

**Conclusions:**

This study addressed important challenges in previous research linking social integration to metabolic health by clarifying the nature and direction of the relationship as it applies to different objectively measured markers and population subgroups. It suggests additional psychosocial and biological pathways to consider in future research on the contributions of social deficits to disease etiology and old-age mortality.

## Background

It has long been believed that the degree to which an individual is embedded in social relations is influential for his or her health and well-being [1-3]. Much evidence has accrued in recent research on the strong associations between social integration and a variety of morbidity and mortality outcomes, such as coronary heart disease [4], malignancies [5], memory loss [6], overall mortality [7], and mortality from vascular diseases [8,9] and cancer [10]. Meta-analyses also suggest that the magnitude of such associations is comparable with that of smoking cessation and exceeds those of many other known risk factors of mortality, such as obesity or physical inactivity [11]. In addition to social, psychological, and behavioral mechanisms, biophysiological processes underlying this relationship have attracted increasing attention, because they can elucidate how social connections “get under the skin” to affect health [12]. Metabolic functions may be particularly important biological pathways, because they are among fundamental physiological determinants of chronic diseases and longevity [13] that are also directly affected by social factors [14,15].

Metabolic syndrome (MetS), defined by a cluster of vascular risk factors that share insulin resistance as a common underlying pathophysiological mechanism [16], is estimated to have affected about 24% of all US adults and 42% of adults aged 70 years and older in the early 1990s [17], and it became more prevalent by 2000 [17]. The metabolic disorders that define MetS, such as abdominal obesity, hypertriglyceridemia, low high-density lipoprotein (HDL) cholesterol, hypertension, and high fasting glucose, are linked to increased risks of arterial disease, diabetes mellitus, and malignancies, all of which are strongly predictive of old-age mortality [14,18,19]. The increasing prevalence and illness burden of metabolic dysregulation in the context of the rising obesity epidemic and the rapid increase of the size of the aging population are thus causes of great concern for public health action.

Social isolation, or the lack of social integration and support, has been increasingly related to physiological functioning in general [12] and metabolic disorders in particular, including higher blood pressure (BP) [20], higher waist to hip ratio [21], glucose intolerance [22], lipid abnormalities, and higher prevalence of metabolic dysregulation [23-25]. Social isolation could lead to adverse biophysiological changes directly as chronic stressors, particularly in older adults and males [7,10]. It also has been shown that social disconnection is associated with various social demographic risk factors for poor metabolic health, such as old age, male sex, nonwhite racial groups, and disadvantaged socioeconomic status [7,9,10]. Furthermore it may influence metabolic risks via behavioral and lifestyle pathways, such as less exercise and more fat intake [26], or through psychosocial pathways, such as perceived loneliness and depressive symptoms [27]. Increased physiological stress response due to a paucity of social ties, as indicated by increased sympathetic nervous system activities, prolonged activation of the hypothalamic-pituitary-adrenal axis, and increased inflammation, plays an important role in the development and exacerbation of hypertension [28] and is critical for metabolism and modulation of immune function [29-31].

A principal limitation of previous studies on the association between social integration and metabolic dysregulation is the use of cross-sectional data that preclude causal inference. The possibility of a reverse causation cannot be ruled out, because physiological stress response, part of the pathogenesis of metabolic disorders, can increase such illness behaviors as withdrawal from social interactions [32]. Second, previous studies often focused on single metabolic markers associated with social relations and produced different results using different markers. A comprehensive assessment of their specific and cumulative effects is lacking. Third, a majority of studies are based on small socially and demographically homogeneous samples that also limit the generalizability of the observed relationships between social connection and metabolic functions. In addition while previous research suggests that the impacts of social ties on MetS are gender specific in Japan [25], it is unknown how such relations may vary across social-demographic subpopulations in the United States.

This study conducted longitudinal analyses of a nationally representative sample of older adults in the United States. It examined the impact of social integration assessed in five waves of data between 1998 and 2006 on six biomarkers of metabolic functions collected in 2006 using latent growth curve models. It also examined whether the associations differ by age, sex, race, and socioeconomic status. In addition to addressing the existing gaps summarized above, this study of older adults facilitates the understanding of the relationship of social factors to aging-related disease, because certain social life events, such as loss of social ties, are more prevalent in late life [33], and the elderly may be more vulnerable to the deleterious effects of social stressors due to declines in adaptive capacities and physiological reserves [34].

## Methods

### Study population

We used data from the Health and Retirement Study (HRS), an openly available nationally representative longitudinal survey of the US population aged 50 years and older conducted every two years from 1992 to 2006 using a multistage sampling of households design with an oversample of black or Hispanic and an overall response rate of about 87%. We used information from noninstitutionalized respondents included in the random one-half of the 2006 sample who consented to the biomarker collection and completed the blood test. Of the 4,435 participants in the biomarker sample in 2006 who had complete biomarker and physical measures included in this study, 112 had missing data on covariates (social integration, demographic and social status, health behaviors, and health status) and were excluded. Compared to those retained in the final sample, those excluded were slightly younger (p < .001), more likely to be male (p < .001) and use cigarettes (p = .014), and less likely to be on hypertension medications (p = .012). They did not differ significantly in any other characteristics or metabolic function measures. Their exclusion thus would not lead to serious selection bias. The final sample consists of 4,323 respondents who had social integration assessments beginning in 1998, the first wave of the HRS with adequate social integration measures, and in the four subsequent waves through 2006.

### Measures

#### Metabolic function

Biomarker measurements were collected by blood spots in the 2006 HRS. The blood test consent procedure, laboratory measures, equipment, and protocols are described elsewhere [35]. The metabolic function was assessed by six biomarkers, including total cholesterol (TC); HDL cholesterol; glycosylated hemoglobin (HbA1c), an integrated measure of blood glucose metabolism over the previous 120 days [36]; waist circumference (WC); and systolic and diastolic BP. For each measure the cutoff points for high risk were defined by clinical practice [16] or empirically defined as the top quartiles (bottom quartile was used for HDL cholesterol). We constructed the index of the overall burden of metabolic dysregulation as the sum of the positive indicators using the two cutoffs. A third alternative we considered was coding those on hypertension or diabetes medications in addition to those above the cutoffs of observed values as the high risk group. We reported results using the quartile cutoffs. This decision was based on both methodological and substantive considerations. Comparing the sample distributions of the six biomarkers suggested that the prevalence of metabolic disorders based on the clinical cutoffs were low in our study population. Additionally, both the clinical and the third categorization including the medicated in the high risk group led to highly inconsistent proportions of at-risk individuals across the multiple metabolic parameters that may complicate the interpretation of the summary index. Substantively, the clinically defined cutoffs may be overly stringent for this community-dwelling sample, whereas the top quartiles are meaningful representations of biochemical abnormalities or less than optimal conditions that may signal predisease pathways. Because the theoretical concept of interest to this study is the actual dysregulation negatively impacted by social disconnection, we considered the actual values observed for various markers to be more relevant than medication status. The same rationale was employed in previous research that suggested that those on medications that actually succeed in lowering BP or cholesterol had lowered levels of these parameters and hence less physiological dysregulation [37]. The high blood pressure (HBP) group combines two measures and includes participants whose systolic BP was greater than 145 millimeters of mercury (mmHg) or whose diastolic BP was greater than 95 mmHg, which are very close to the clinical cutoff points for hypertension. The cutoff points for HDL cholesterol and WC were sex-specific based on previous literature [16,38]. The final metabolic dysregulation score ranges from 0 to 5.

#### Social integration

Based on the widely used Berkman social network index (SNI) and a previous study of the HRS data [6], we assessed the degree of social integration by summarizing the number of social ties across five domains of social activities: marital status, contact with parents, contact with child(ren), contact with neighbors, and volunteer activities. Each of these ties is a dichotomous variable indicating the presence or absence of social integration and was set to missing if the respondent was missing data for that domain. Marital status was coded 1 if respondents were currently married and 0 otherwise. Respondents were asked how often they had contact with their parents (including mother, father, mother-in-law, and father-in-law) and child(ren) (including child[ren]-in-law and stepchild[ren]) either in person or by phone or mail and how often they had gotten together with neighbors just to chat or for a social visit in the last year. The indicators of contact with parent, child(ren), and neighbors were each coded 1 when respondents reported more than one time per week to the corresponding questions and 0 otherwise. Respondents without living parents or child(ren) were coded as 0 for the corresponding domain. The volunteer status was coded as 1 if respondents reported ever having volunteered for religious, educational, health-related, or other charitable organizations in the past 12 months and 0 otherwise. The social integration score is the sum of nonmissing scores from the five dichotomized variables and ranges from 0 to 5.

We compared the use of the social integration score as a continuous versus a categorical variable in preliminary analyses. We dichotomized the score into high integration (1 if score > 3) and low integration (0 if score ≤ 3) in the final analyses, because it better captures the nonlinear or threshold effect and yields a substantially better model fit.

#### Covariates

We adjusted for factors reviewed earlier that have been associated with both social relationships and biomarkers of metabolic functioning that may confound the associations of interest to this study. Specifically, these include demographic and social status as indicated by age, sex, race, education (years of schooling), and household income (total income of self and spouse only); health behaviors as indicated by cigarette smoking status, excessive drinking (defined as > 12 days of four or more drinks per occasion in the last three months), and regular exercise (defined as ≥ 1 times per week); and health status as indicated by body mass index (BMI), depressive symptoms (a modified version of the Center for Epidemiologic Studies Depression Scale [CES-D]), the number of diagnosed chronic disease conditions (including cancer, chronic lung disease, heart disease, stroke, psychiatric problems, and arthritis), and whether the respondents were on hypertension medication and/or diabetes medication. All but three covariates were assessed at baseline. The measure of household income is complete across the entire sample only in 2006. To ensure comparability of economic status among all respondents, we used the 2006 income distribution to define relative status in quartiles. The BMI and medication variables were only recorded at the final wave in 2006.

##### Statistical analysis

**Figure 1:**
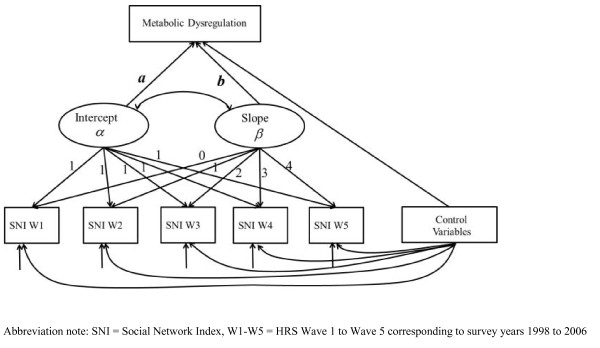
Representation of the latent growth curve model and hypothesized relationships between social integration and metabolic dysregulation.

We conducted descriptive analyses to examine bivariate correlations between social integration at baseline and biomarkers measured in 2006 and covariates at baseline using the t-test for continuous variables and the chi-squared test for categorical variables. We then used the latent growth curve models to ascertain the impact of social integration assessed over the five waves of surveys spanning the eight years from 1998 to 2006 on the risks of metabolic disorders assessed by the individual markers and the overall burden of metabolic dysregulation in 2006. The latent growth curve models enabled us to most effectively use the multiwave data on social integration and facilitated the longitudinal analysis crucial for causal inference of its effect on metabolic function over time. As summarized in Figure 1, the model assumes that the social integration or SNI score for an individual at a specific point in time is determined by an underlying linear process or latent trajectory [39,40]. It can be characterized by two latent parameters, the intercept and the slope, which stand for the initial status and the growth rate of the process, respectively. That is, the intercept measures the mean baseline level of social integration, and the slope characterizes the average change of social integration with time. The specific hypothesis we examined using this model is how the longitudinal trajectories of social integration defined by the mean level and the change of social integration from wave 1 to wave 5 affect individuals’ metabolic function at wave 5 through the estimation of the coefficients a and b.

We used continuous variables of metabolic markers and the summary index in linear models. And we used the dichotomous variables of metabolic markers in logistic models and the categorical variable of the summary index in ordinal logit models. We reported results from the latter models because of their vastly superior model fits based on the Bayesian information criterion. We also grouped those with summary index scores of 3 to 5 into one category, because it improved the model fit.

We adjusted for baseline covariates in all models to control for the long-term effects of these factors on the outcomes. Due to the unavailability of measures of income, BMI, and medication use at baseline, we used these variables measured in 2006 instead. Additional analyses and modeling considerations suggest that this restriction did not weaken the results about the prospective associations of interest. First, because the study population was comprised of older adults near or past retirement age, the relative income status was quite stable over time, as suggested by additional analysis comparing the final sample with the smaller sample with incomplete income data at baseline. Income levels were also highly consistent with education levels that did not vary during the study period. Second, both BMI and medications were correlated with some chronic conditions and health status variables at baseline. The inclusion of all these baseline covariates should effectively control for potential prior health effects on social integration over time. Third, the metabolic markers being examined could be much more highly confounded with contemporaneous than prior status of BMI and medication use. For instance, the measures of central obesity and BP could be directly affected by one’s overall obesity and HBP medications at the same point in time. Adjusting for the latter can thus take into account current management of medical conditions.

For each metabolic outcome variable, we first estimated basic models with controls of only age, sex, race, and BMI and then estimated the fully adjusted models with controls of all other covariates. The vast majority of covariates was statistically significant at the α < .05 level in the full models and were retained. The removal of nonsignificant covariates did not change the results regarding remaining covariates but improved the model fit, suggesting that the final models are more parsimonious and better summaries of relevant risk factors for each outcome. To assess how the metabolic effects of social integration vary across subpopulations, we estimated these two models in subsamples stratified by age (< 65 years vs. 65 years and older), sex, race, education (< 12 years vs. 12 years or more), and household income (lower quartiles vs. top quartile). For all analyses we used the 2006 biomarker sample weights (supplemented to the core sample weights) to account for the complex sample design and nonresponse [41].

## Results

Sample characteristics of the respondents are shown in Table 1, which includes the descriptive statistics of the metabolic outcomes measured in 2006 and other covariates measured at baseline for the sample as a whole and also by social integration level at baseline. About 26.3% of the sample respondents reported a high level of social integration (scores 4–5). Metabolic functions marked by HbA1c, TC, and systolic BP were significantly worse for those with low social integration than those with high social integration, as indicated by the p values for significance tests of the bivariate associations. The overall burdens of metabolic dysregulation were also significantly higher for those with low social integration, as shown by their lower proportions of 0–1 counts and higher proportions of 2–5 counts of the overall metabolic dysregulation score (p < .001). A few other characteristics show significant differences across respondents in different social integration levels. Compared to respondents with high social integration, those with low social integration were older, more likely to be nonwhites, less well educated, less likely to be in the top income quartile, more likely to be smokers, had no regular exercise, were more depressed, had more chronic conditions, and were more likely to be on hypertension medication. This is preliminary evidence that the associations of social integration with metabolic functioning are partially due to differences in the covariates included.

**Table 1:** Sample characteristics by social integration at baseline, Health and Retirement Study, 1998–2006

**Variable**	**All**	**Low social integration (0–3)**	**High social integration (4,5)**	**Difference p value**
	**(N = 4,323)**	**(N = 3,184)**	**(N = 1,139)**	
**Metabolic function (2006), mean (SD)**				
TC (mg/dl)	200.6 (40.5)	199.7 (40.2)	202.9 (41.0)	0.025
HDL cholesterol (mg/dl)	57.7 (14.6)	57.4 (14.2)	58.2 (15.6)	0.156
HbA1c (%)	5.8 (0.8)	5.8 (0.8)	5.7 (0.8)	0.004
WC (mm)	100.5 (14.4)	100.7 (14.4)	100.1 (14.6)	0.278
BP				
Systolic BP (mmHg)	130.6 (19.9)	131.7 (20.3)	127.6 (18.1)	< 0.001
Diastolic BP (mmHg)	80.1 (11.2)	80.2 (11.5)	79.9 (10.4)	0.421
Metabolic dysregulation index				
0	25.6%	24.3%	29.1%	< 0.001
1	35.7%	35.0%	37.6%	
2	25.3%	26.8%	21.4%	
3–5	13.4%	14.0%	12.0%	
**Demographic and social status**				
Age, mean (SD)	53.7 (10.0)	54.7 (10.3)	51.2 (8.8)	< 0.001
Sex, 1 = female (%)	54.9%	55.2%	54.1%	0.510
Race, 1 = nonwhite (%)	13.5%	15.1%	9.2%	< 0.001
Education, 1 = 12+ years (%)	50.9%	47.0%	61.3%	< 0.001
Household income, 1 = top quartile (%)	25.0%	19.7%	38.9%	< 0.001
**Health behaviors**				
Cigarette smoking, 1 = ever (%)	56.3%	57.9%	51.8%	< 0.001
Excessive drinking, 1 = yes (%)	1.9%	2.0%	1.7%	0.472
Regular exercise, 1 = yes (%)	46.2%	43.9%	52.3%	< 0.001
**Illness/conditions**				
BMI, mean (SD)	29.2 (5.7)	29.1 (5.8)	29.5 (5.7)	0.309
Depressive symptoms (CES-D), mean (SD)	1.4 (1.8)	1.5 (1.9)	1.0 (1.5)	< 0.001
Chronic conditions, mean (SD)	0.7 (0.8)	0.8 (0.8)	0.6 (0.7)	< 0.001
On HBP medications (%)	46.1%	47.2%	43.1%	0.017
On diabetes medications (%)	13.9%	14.4%	12.6%	0.139

Note: Weighted statistics for demographic and social status, health behaviors, and illness variables are reported for baseline except for household income, BMI, and medications, which are only available in 2006. SD = standard deviation; mg/dl = milligrams per deciliter; mm = millimeters.

**Figure 2:**
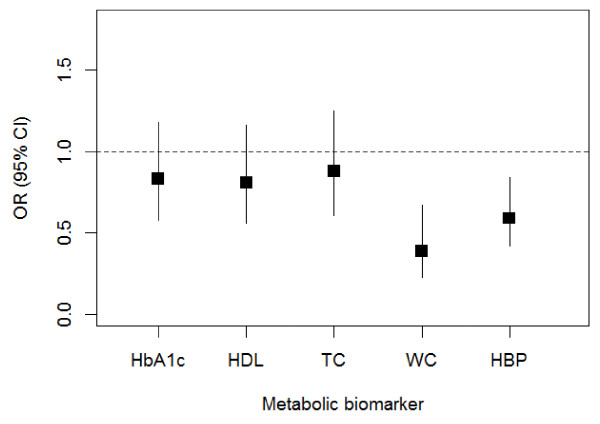
Estimated mean effects of social integration (modeled as latent growth curves) on individual metabolic risk factors.

We first examined social integration modeled as a latent growth curve in relation to individual markers of metabolic disorders. Figure 2 presents the mean estimates (of coefficient a in Figure 1) in terms of the odds ratios (ORs), adjusting for demographic covariates and BMI. Having a high mean level of social integration decreased the odds of high risk HbA1c, HDL cholesterol, TC, WC, and HBP at a later point in time, other things being equal. The ORs are statistically significant for WC and HBP and indicate a 61% (OR [95% confidence interval {CI}] = 0.39 [0.23, 0.67], p = .007) and 41% (OR [95% CI] = 0.59 [0.42, 0.84], p = .021) reduction in the risks of these two disorders, respectively. Adjusting for all other covariates slightly reduced the ORs, but the protective effects of high social integration remained substantial and significant. Stratified analyses by social and demographic subgroups show similar results, with social integration having more significant effects for whites and the low-income group. The slope estimates (of coefficient b in Figure 1) were not statistically significant at the α < .05 level for any markers, suggesting no effect of change in social integration over time on metabolic disorders at a later time. Our preliminary analyses show that individual trajectories of social integration were relatively stable and constant. In this case the mean level subsumes most of the social integration effects on these markers such that the slope estimates do not provide much additional information above and beyond the mean estimates.

Table 2 presents the ordered logistic model estimates of the effects of social integration on the summary index of metabolic dysregulation. Similar to the results above for individual markers, we present only the mean estimates, as few slope estimates were statistically significant. For the sample as a whole, model 1, adjusting for age, sex, race, and BMI, shows an OR of 0.54 (95% CI = [0.40, 0.72]) that is highly significant (p < .001). The effect is slightly smaller (OR [95% CI] = 0.63 [0.46, 0.86]) in model 2, which adjusts for all the other covariates but remains substantial in size and statistically significant (p = .002). These results suggest that having a high mean level of social integration vastly reduced the overall burden of metabolic dysregulation at a later point in time. While socioeconomic status, health behaviors, and illness and medications can account for a small portion of such effect, the independent effect of social integration remains strong.

**Table 2:** Estimated mean effects of social integration (modeled as latent growth curves) on the index of metabolic dysregulation

	**Model 1**		**Model 2**	
	**OR (95% CI)**	**P value**	**OR (95% CI)**	**P value**
**All** (N = 4,323)	0.54 (0.40, 0.72)	< 0.001	0.63 (0.46, 0.86)	0.002
**Age**				
< 65 years (N = 1,697)	0.47 (0.29, 0.75)	0.002	0.57 (0.34, 0.95)	0.030
65 or more years (N = 2,626)	0.65 (0.47, 0.90)	0.010	0.74 (0.53, 1.05)	0.090
**Sex**				
Male (N = 1,714 )	0.53 (0.31, 0.91)	0.020	0.58 (0.33, 1.02)	0.059
Female (N = 2,609)	0.50 (0.35, 0.73)	< 0.001	0.64 (0.44, 0.96)	0.029
**Race**				
White (N = 3,597)	0.52 (0.38, 0.71)	< 0.001	0.62 (0.45, 0.86)	0.004
Nonwhite (N = 726)	0.50 (0.20, 1.24)	0.133	0.61 (0.23, 1.64)	0.328
**Education**				
< 12 years (N = 2,333)	0.50 (0.32, 0.79)	0.003	0.53 (0.33, 0.84)	0.007
12 or more years (N = 1,990)	0.64 (0.42, 0.97)	0.034	0.72 (0.46, 1.13)	0.154
**Household income**				
Lower quartiles (N = 3,252)	0.58 (0.40, 0.84)	0.004	0.63 (0.43, 0.93)	0.032
Top quartile (N = 1,071)	0.65 (0.37, 1.14)	0.132	0.64 (0.37, 1.08)	0.090

Note: Model 1 adjusted for age, sex, race, and BMI; model 2 adjusted for all covariates.

The protective effect of high social integration holds across most social demographic subpopulations but also shows slight variations. Table 2 indicates that having a high mean level of social integration had a more pronounced impact on the younger old, or respondents under age 65 (model 1: OR [95% CI] = 0.47 [0.29, 0.75]), as compared to adults above age 65 (OR [95% CI] = 0.65 [0.47, 0.90]). Adjusting for all covariates reduced the ORs for both in model 2, but that for the younger old remains statistically significant. The social integration effect is similar for male and female respondents in model 1 but is larger for males than females in model 2. The less significant effect for males suggests more variation in the mean effect within the male sample. The ORs are significant for whites but not for nonwhites. This could be due to the much smaller sample size for the racial minorities that reduced the statistical power for the models based on this subsample. The protective effect of social integration is larger and more statistically significant for those with less than a high school education than for those with high school diplomas or more both without and with adjustments of all the other covariates. And the effect is also largely restricted to those in the lower-income quartiles as opposed to those in the top quartile. The comparison of ORs estimates in these models also suggests that adjusting for social and health behavior–related factors reduced the effects of social integration more for older adults than younger old adults, females than males, the better educated than the less well educated, and those with lower than higher incomes.

## Discussion

This study addressed some important challenges in previous research on social relations and metabolic health. First, what is the nature and direction of the relationship between social integration and metabolic dysregulation? Our findings are based on longitudinal analyses of the largest nationally representative sample of the elderly US population. The assessments of social network characteristics in the HRS occurred in multiple time points prior to the assessment of biomarkers of metabolic function, thus allowing us to use the prospective information on social integration to estimate its longitudinal trajectories in relation to metabolic variables over time. The latent growth curve analyses were therefore unlikely to be affected by reverse causation. We found evidence that a high level of social integration predicted lower odds of metabolic disorders, such as abdominal obesity and hypertension, and also lower odds of overall metabolic dysregulation in a large sample of over 4,000 community-dwelling adults age 50 and above followed over eight years. Being highly socially integrated, that is, having four to five social ties (with spouse, family, or neighbors or volunteering with a social organization), cut the risk of metabolic dysregulation by close to one-half, on average. And stably high social integration had more potent metabolic impacts over time than changes therein. In all, our study indicates that metabolic dysregulation may be one major physiological mechanism through which social embeddedness is related to morbidity and mortality in old age.

Second, does the relationship between social integration and metabolic function apply to different markers? This question is relevant for broadening the understanding of metabolic response to social stressors and future investigations of specific biological pathways. Previous studies are limited in the number of metabolic markers assessed simultaneously in the same analyses. Yet each marker reflects different dimensions or stages of metabolic changes. And the existence or clustering of multiple risk factors for a given individual may present distinct metabolic challenges [42]. The possibility that individual markers and cumulative burden may bear different relationships to social integration was not tested. Our study shows the advantage of investigations of both multiple metabolic markers and the summary index of metabolic functions. The analysis of five individual metabolic disorders as outcomes suggests that social integration had differential impacts on distinct aspects of fat and glucose metabolism, with the protective effect being particularly salient for larger WC and HBP. The composite measure of metabolic dysregulation further captures the presence of multiple individual risk factors and hence overall burden of metabolic disorders, much the same way that the allostatic load reflects the cumulative dysregulation across multiple physiological systems [31]. Our analysis shows that the summary index has considerable power in accounting for metabolic effects of social relations. Taken as a whole, these results suggest the importance of considering different physiological pathways in further analysis of the interplay between social and biological factors in disease etiology.

Furthermore we asked the question whether the association varies by individual characteristics that define major social and demographic statuses. The most interesting finding is that the social integration and metabolic function links were consistent across most subpopulations by age, sex, race, and socioeconomic status, with the reductions in the risks ranging between 40% and 50% for individual markers and the overall index of metabolic dysregulation. Results of this degree represent the very real benefits of social integration on major public health indicators that transcend demographic and socioeconomic strata. The constant effect size across participant characteristics, then, suggests that the protective effects of social integration against metabolic risks may be generalized and that efforts to reduce such risks should not be isolated to subgroups.

In this context our study also reveals some population heterogeneity in the relationships between social integration and metabolic functions that may reflect differential exposures or susceptibilities to social stressors and metabolic disorders across subgroups. Specifically, we found that the link was more salient for the younger old adults (under age 65), males, and the socioeconomically disadvantaged. In contrast to the age pattern in the links between social integration and markers of inflammation and MetS that suggests stronger associations in older adults [10,14], this study shows a slightly stronger effect of social integration on metabolic dysregulation in younger old adults. The difference may be due to the age composition of the samples in that the former studies included adults of all ages, whereas ours consists of only adults above age 50. It may also suggest age-specific links between social integration and different physiological markers (inflammation vs. metabolic function) that warrant attention in future studies. The larger residual effect of social integration for males, after the adjustment of an array of social and behavioral factors, is consistent with a previous study that found greater male inflammatory response to social isolation in US adults [14] and may reflect sex-specific stress reactivity patterns that evolved as adaptations to different social and biological roles, which are well documented in animal studies [43]. The larger protective effect of social integration for the less educated and those with lower incomes could result from a resource substitution process whereby individuals who were lacking or disadvantaged in socioeconomic and related resources substitute engagement in social network ties that confer physiological benefits for those resources [44]. This hypothesis, however, is not supported by the finding on race variation in that there is no evidence of any effect of social integration for nonwhites, who generally have lower socioeconomic statuses. While this may be a function of the small sample size of the racial/ethnic minority group in the current dataset, other unmeasured social and physiological mechanisms could be involved and need to be more fully investigated in future research.

Several study limitations can be identified that invite additional research. First, the measure of metabolic dysregulation largely resembles but is not identical to that of the MetS commonly used in clinical settings. Two of the consisting markers for the original MetS, namely, fasting glucose and triglycerides, are not available in the HRS biomarker study, although a more stable and longer-term measure of glucose tolerance as measured by HbA1c was substituted for the former. In preliminary analyses we experimented with the alternative operationalization that more closely replicated that of the MetS by dichotomizing the score (those having three or more risk factors being coded as 1). The results are similar to those presented in the final analysis that used the summary count score, with the latter showing a superior model fit. For these reasons we did not precisely replicate the operationalization of the MetS but were able to capture the same clustering of risk factors encompassing fat and glucose metabolism. The findings regarding the summary index of metabolic dysregulation should nonetheless be interpreted based on the available markers only instead of as being equivalent to the MetS.

Second, social relationships are inherently a multidimensional construct. Due to the data limitations, this study focused only on the quantitative and objective dimension, namely, social network size, that is most often studied. Qualitative or subjective appraisals of social relations, such as perceived social support or satisfaction with support, could also be important. Whether they would also manifest strong if not stronger effects on metabolic health as measured in this study is not clear. When simultaneously included in recent studies of other health outcomes, the number of social network ties was found to be more significantly related to disease and mortality than perceived social support [9] or loneliness [7]. It was also suggested that the direct effects of social isolation are partly mediated by perceived loneliness [7]. Future studies including multiple types and characteristics of social relationships would enrich our understanding of their multifaceted links to physical health, their interrelations, and their relative importance to different outcomes.

Third, the finding that the metabolic influence of social connections cannot be completely explained by social and health behaviors included in the study or other psychosocial processes in previous studies mentioned above [7,10] suggests that social connections exert an independent effect that needs further explanation. While the descriptive analyses suggest apparent differences in many characteristics across the social integration strata that are consistent with findings from prior research, there may well be other unmeasured confounding factors, such as the relationship quality discussed above, that were not included in the present study. There may also be multiple biological pathways, such as cardiovascular, endocrine, and immune systems, involved in the metabolic response to social relationships that can be intertwined with behavioral and psychological factors. Given the vast array of determinants of fat and glucose metabolism, additional research is needed to examine the mechanisms by which social relationship deficits contribute to metabolic disorders. Furthermore although the lack of baseline measures of several covariates was not particularly problematic for the associations under investigation in this study, longitudinal measures of exposures or risk factors will substantially strengthen causal inference should they become available in future studies. In addition interdisciplinary perspectives and integrations of social and biological data will become more important than ever in future studies of population health processes given the complicated nature of the phenomenon.

## Conclusions

The metabolic disorders evaluated in this study may mark the onset and/or progression of numerous aging-related conditions, most notably cardiovascular disease and diabetes, which are increasingly the primary causes of morbidity, functional disability, and mortality in industrialized nations. Our study clearly indicates that social integration or the lack thereof largely affects risks of metabolic dysregulation in aging adults, who typically experience rapid declines in physiological resilience. And demographic trends, such as decreasing household size and increasing geographic mobility, coupled with higher rates of widowhood and loss of relatives and friends in late life place older adults at greater risk of social isolation and loneliness [45]. The synthesis of the longitudinal relationship between social integration and metabolic outcomes together with the clarification of the consistency or heterogeneity in such associations in subpopulations are the major contributions the present study has made and may be particularly informative to public health actions, clinical practice, and policies aimed at reducing social and behavioral risks for morbidity and mortality. Given the importance of metabolic functions for both quality and quantity of life, continued efforts at developing efficacious interventions, such as those targeted at enhancing social connections among older adults, have the potential for sizable yields in the arena of healthy longevity.

## Abbreviations

BMI: Body mass index; BP: Blood pressure; CES-D: Center for epidemiologic studies depression scale; CI: Confidence interval; HbA1c: Glycosylated hemoglobin; HBP: High blood pressure; HDL: High-density lipoprotein; HRS: Health and retirement study; MetS: Metabolic syndrome; mg/dl: milligrams per deciliter; mm: millimeters; mmHg: millimeters of mercury; OR: Odds ratio; SD: Standard deviation; SNI: Social network index; TC: Total cholesterol; WC: Waist circumference.

## Competing interests

The authors declare that they have no competing interests.

## Authors’ contributions

YCY, lead author, originated ideas, designed the study, directed statistical analyses, and wrote the manuscript. TL, coauthor, assisted with the literature search and data preparation, performed the statistical analyses, and contributed to the interpretation of results. YJ, coauthor, conducted the initial literature review and prepared data for statistical analyses. All authors read and approved the final manuscript.

## Pre-publication history

The pre-publication history for this paper can be accessed here:

http://www.biomedcentral.com/1471-2458/13/1210/prepub
